# Recent Researches on Hæmatozoa

**Published:** 1887-04

**Authors:** Wm. Osler


### EDITORIAL DEPARTMENT.

### RECENT RESEARCHES ON HÆMATOZOA.

BY WM. OSLER.

Blood parasites belonging to the Nematoda and Trematoda have long been known and certain filarian forms are very widely distributed. Recent observations indicate that the blood of many species of animal is infested with parasitic protozoa, some of which are probably pathogenic. Our accurate knowledge on this subject dates from the work of Mitrophanow, in 1883, (Biol. Centralblatt Bd. iii) who described in the mudfish and carp, organisms which, with a certain resemblance to small nematodes, proved, however, to be flagellate infusoria. Each consisted of a highly contractile proplasmic body, a spiral membrane and a flagellum. There were also forms without membrane or flagellum which presented amæboid movements. Mitrophanow regarded these organisms as infusoria, between the genera Cercomonas and Trichomonas and suggested a new genus, Hæmatomonas. In 1879 Lewis described (Quarterly Jr. Mic. Science 1879) in the blood of rats in India, mobile filaments which he thought at first were spirilla, but which he subsequently determined to belong to the protozoa. They were present in 29 per cent of Indian rats. Quite recently, Crookshank has investigated the subject (Journal of the Royal Microscopical Society 1886) and finds these organisms in 25 per cent of English rats. He describes them as polymorphic with slightly tapering bodies which terminate at one end in a stiff, immobile, spine-like process and at the opposite have a long flagellum, while longitudinally attached there is a delicate undulating fin-like membrane. There are various other forms, globose, angular, semi-circular and disc-shaped, representing phases in the development of this organism, which Crookshank refers provisionally to the genus Trichomonas.

In the Archives Slaves de Biologie 1886 (Abstract in Centralblatt f. d. Med. Wissenschaften 1886), Danilewsky describes hæmatozoa in birds, particularly water fowl, which have a close resemblance to those of the fish and the rat. Certain of the forms however occur within the red blood corpuscle, and the organism has amæboid and flagellate phases of existence.

So far as can be ascertained the existence of these parasites in fish, birds and in the rat in no way interferes with health, so that they can scarcely be regarded as pathogenic.

A practical interest in this subject has been aroused by the discovery of hæmatozoa of this description in the blood of animals and man affected with certain diseases. In 1880 Dr. Griffith Evans, described in horses, mules and camels in India, a disease called *surra*, which was exceedingly fatal in the Punjab. The affection is characterized by fever, jaundice, hemorrhages, rapid wasting, and death in from two weeks to two months. The Third Punjab Cavalry lost 300 horses in the epidemic. Evans found a parasite in the blood and was able to induce the disease in healthy animals by inoculation. He regarded the organism as a spirillum but subsequently concluded that it did not belong to the bacteria. In 1884 the *surra* disease broke out in Burmah in the transport mules, and Veterinary Surgeon Steele made an exhaustive investigation of the epidemic, and an elaborate report has been issued by the

Punjab government. He describes the disease as a true relapsing fever and regards the organism in the blood as a spirillum, similar in character to that of the recurrent or relapsing fever of man, and named it *Spirochæta Evansi.* Recently on his return from India, Dr. Evans placed his preparations in the hands of Dr. Crookshank, who had no difficulty in determining that the parasites in the blood were not schizomyces, but protozoa very similar to, if not morphologically identical with,the flagellate organisms described by Lewis in the blood of the rat. Both Evans and Steele regard the parasite as pathogenic, and when the disease is inoculated they occur in the blood in large numbers. Steele also determined that it was present during the fever and absent in the apyrexial periods.

Five or six years ago Laverau, a French army surgeon, stationed in Algiers, found certain bodies in the blood of patients affected with malaria. The observation of over 400 cases enabled him to assert that they were constant, specific and distinctive. There were several forms: 1st, a pigmented body in the interior of the red corpuscle which presented slow amæboid changes; 2d, crescentic bodies; 3d, free pigmented forms and 4th, flagellate bodies. These different forms he regarded as phases in the development of a parasite. These observations have been confirmed by Richard and more recently by Marchiafava and Celli in Italy, and by Councilman and myself in the United States. The description of the bodies by these various workers in different parts of the world is practically identical. The different forms are phases in the life history of a single organism and there can be no doubt that it too belongs to the flagellate protozoa. Marchiafava and Celli have called it the *plasmodium malariæ*, but I have suggested (British Medical Journal, March 12, 1887) that it be placed in Mitrophanow's genus *Hæmatomonas,* where it evidently belongs. Danilewsky calls attention to the similarity of the form he has described in birds to those which occur in malaria, and suggests that they are identical. They appear to be constant in the blood of persons affected with the different forms of ague and so far have not been found under other conditions. Whether pathogenic or not has not yet been determined.

